# Dr. Hiram Dana Walker

**Published:** 1913-04

**Authors:** 


Dr. Hiram Dana Walker, Buffalo 1864, died at his home in Buffalo Feb. 21, aged 73. He was a member of the A. M. A. and its branches and of the Buffalo Academy of Medicine. For many years he had devoted his time to the study of cancer, which he believed to be due to a parasite propogated by the earth-worm. While his theory was not generally accepted, all who knew him were impressed with his sincerity and with the amount of study which he had devoted to the subject. In scientific research, especially along lines having so practical and humanitarian interest, equal credit must be given to those whose labors have resulted negatively, especially in regard to subjects whose final solution has not been accomplished.

